# FOXO1 regulates pentose phosphate pathway-mediated induction of developmental erythropoiesis

**DOI:** 10.3389/fcell.2022.1039636

**Published:** 2022-10-12

**Authors:** Anuntxi Monsalve, Isaac Canals, Leal Oburoglu

**Affiliations:** ^1^ Molecular Medicine and Gene Therapy, Lund Stem Cell Center, Lund University, Lund, Sweden; ^2^ Neurology, Lund Stem Cell Center, Lund University, Lund, Sweden

**Keywords:** developmental hematopoiesis, pentose phosphate pathway, FOXO1, endothelial to hematopoietic transition, erythropoiesis

## Abstract

Primitive, neonatal and adult erythroid cells have been previously shown to have an active pentose phosphate pathway (PPP) that fuels various processes. However, it is unclear whether the PPP plays a role during the emergence of erythroid progenitors from hemogenic endothelium (HE). In this study, we explored PPP and its genetic regulation in developmental erythropoiesis. We induced hematopoietic differentiation of human induced pluripotent stem cells (hiPSCs) to obtain HE cells. These cells were treated with lentiviral vectors harboring shRNAs against FOXO1, or with inhibitors against the PPP, NRF2 or AKT. Erythroid differentiation, proliferation and frequency were evaluated by flow cytometry. Gene expression was assessed by qPCR or by analysis of available RNAseq data. We found that PPP is indispensable for the erythroid differentiation of HE cells and it partially fuels nucleotide biosynthesis. Moreover, we showed that NRF2 and AKT are essential, while FOXO1 is detrimental, for HE-derived erythroid differentiation. In contrast, blocking FOXO1 expression did not affect erythroid differentiation of cord-blood HSPCs. Mechanistically, FOXO1 inhibition in HE cells led to an increase in the non-oxidative branch of the PPP. During developmental erythropoiesis, the gradual decrease in FOXO1 activates the PPP and fuels nucleotide biosynthesis and cell proliferation.

## Introduction

Red blood cells (RBCs), the most abundant cell type in the bloodstream and in the human body, are endowed with the ability to carry and deliver oxygen to body tissues and organs. These cells have been extensively studied both during embryonic development and in adult organisms. During development, erythroid cells arise through three hematopoietic waves that occur at distinct developmental stages. Specifically, a first wave of primitive hematopoiesis gives rise to primitive erythroid cells in the yolk sac (Palis et al., 1999), followed by a second definitive wave which results in erythro-myeloid progenitors (EMPs) among other cells (Chen et al., 2011), and a third wave during which hematopoietic stem cells (HSCs) emerge (Medvinsky and Dzierzak, 1996; Ivanovs et al., 2011) and possess the potential to differentiate into definitive erythroid cells. During all three waves, hematopoietic cells emerge from endothelial cells with hemogenic potential termed hemogenic endothelial cells (HE) (Choi et al., 2012; Frame et al., 2016; Stefanska et al., 2017) through endothelial to hematopoietic transition (EHT) (Boisset et al., 2010; Kissa and Herbomel, 2010).

Even though they lack mitochondria and nuclei, RBCs preserve several metabolic pathways to properly function, including glycolysis, adenosine metabolism, the Rapoport-Leubering shunt and the pentose phosphate pathway (PPP) (Wiback and Palsson, 2002). In fact, the first enzyme of the PPP, glucose 6-phosphate dehydrogenase (G6PD), was initially studied in RBCs as genetic mutations in the *G6PD* gene leads to hemolytic anemia (Kowalik et al., 2017). Previously, we showed that the PPP is indispensable for erythroid differentiation of umbilical cord blood (UCB)-derived hematopoietic stem and progenitor cells (HSPCs) (Oburoglu et al., 2014). Moreover, recently, PPP activity was shown to increase during the maturation of primitive erythroid cells in the mouse embryo, specifically during the transition from basophilic to orthochromatic erythroblasts (Nemkov et al., 2022). Nevertheless, it is still unclear whether the PPP is required during the first emergence of human hematopoietic cells in the embryo, namely when hemogenic endothelium (HE) gives rise to primitive erythroid progenitors.

While investigating the proliferation rate of iPSC-derived HE cells during hematopoietic differentiation, we previously found that the fastest proliferating HE-derived cells were erythroid progenitors (Oburoglu et al., 2021). As rapidly proliferating cells require ample amounts of nucleotides, we hypothesized that the ribose-producing PPP could be essential for erythroid differentiation. Indeed, the PPP converts glucose to ribose-5-phosphate, the precursor for the ribose component of nucleotides (Voet and Voet, 2011). The PPP has two arms: an oxidative arm [involving glucose-6-phospahte dehydrogenase (G6PD)] and a non-oxidative arm [with transketolase (TKT)], both of which can lead to the production of ribose-5-phosphate (Tong et al., 2009). Here we show that PPP activity and nucleotide biosynthesis are indispensable for erythroid differentiation of human HE cells, which is controlled by AKT and NRF2. We further dissect the use of PPP during this process and uncover an essential role for FOXO1 during developmental erythropoiesis.

## Results

### The pentose phosphate pathway fuels hemogenic endothelium-derived erythroid cell proliferation

**FIGURE 1:**
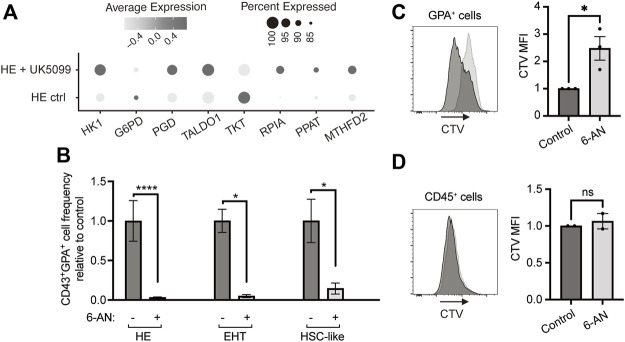
Blocking the pentose phosphate pathway inhibits HE-derived erythroid cell proliferation and output. **(A)** Dot plots show expression levels of PPP enzymes for HE ctrl and HE + UK5099 in hematopoietic clusters from our previously published dataset, as detected by scRNAseq and based on percent expressed (dot size) and average level of expression (color intensity). **(B)** iPSC-derived HE, EHT and HSC-like cells were subcultured with 25 µM 6-AN and bar graphs show CD43^+^GPA^+^ cell frequency ± SEM relative to the untreated control on day 6 (*n* = 5 for HE cells and *n* = 3 for EHT and HSC-like cells, paired *t*-tests). Culture media was changed every 2 days and inhibitors were added at every media change. **(C,D)** iPSC-derived GPA^+^ cells **(C)** or CD45^+^ cells **(D)** were sorted on day 13 of hematopoietic differentiation, stained with CellTrace Violet (CTV) dye and treated with 25 µM 6-AN. The CTV dye is diluted with each division and therefore allows to assess proliferation. Mean fluorescence intensity of CTV was assessed by flow cytometry 3 days later. Representative plots and bar graphs ± SEM are shown (*n* = 3 for GPA^+^ and *n* = 2 for CD45^+^ cells, paired *t*-tests). ns, not significant, *p < 0.05, ****p < 0.0001.

We sought to evaluate the requirement for PPP in developmental erythropoiesis and assessed the expression of PPP enzymes in our previously published single-cell RNA sequencing (scRNAseq) dataset (Oburoglu et al., 2022). Under conditions where HE cells were pushed towards a primitive erythroid fate with the mitochondrial pyruvate carrier (MPC) inhibitor UK5099, the expression of six out of eight main enzymes of PPP were upregulated (Figure 1A). To understand whether PPP is required during erythroid differentiation from iPSC-derived HE, EHT and HSC-like populations, we treated these cells with a G6PD inhibitor, 6-aminonicotinamide (6-AN). Strikingly, the frequency of CD43^+^GPA^+^ erythroid cells deriving from HE, EHT or HSC-like cells was significantly reduced at day 6 following 6-AN treatment (Figure 1B). The PPP produces ribose-5-phosphate for nucleotide biosynthesis, therefore we investigated whether blocking the PPP affects cell proliferation. We used the CellTrace Violet dye to track divided GPA^+^ erythroid cells and found that 6-AN treatment significantly blocked cell proliferation after 3 days of culture (Figure 1C). However, we did not see this effect in 6-AN-treated CD45^+^ non-erythroid hematopoietic cells (Figure 1D).

**FIGURE 2:**
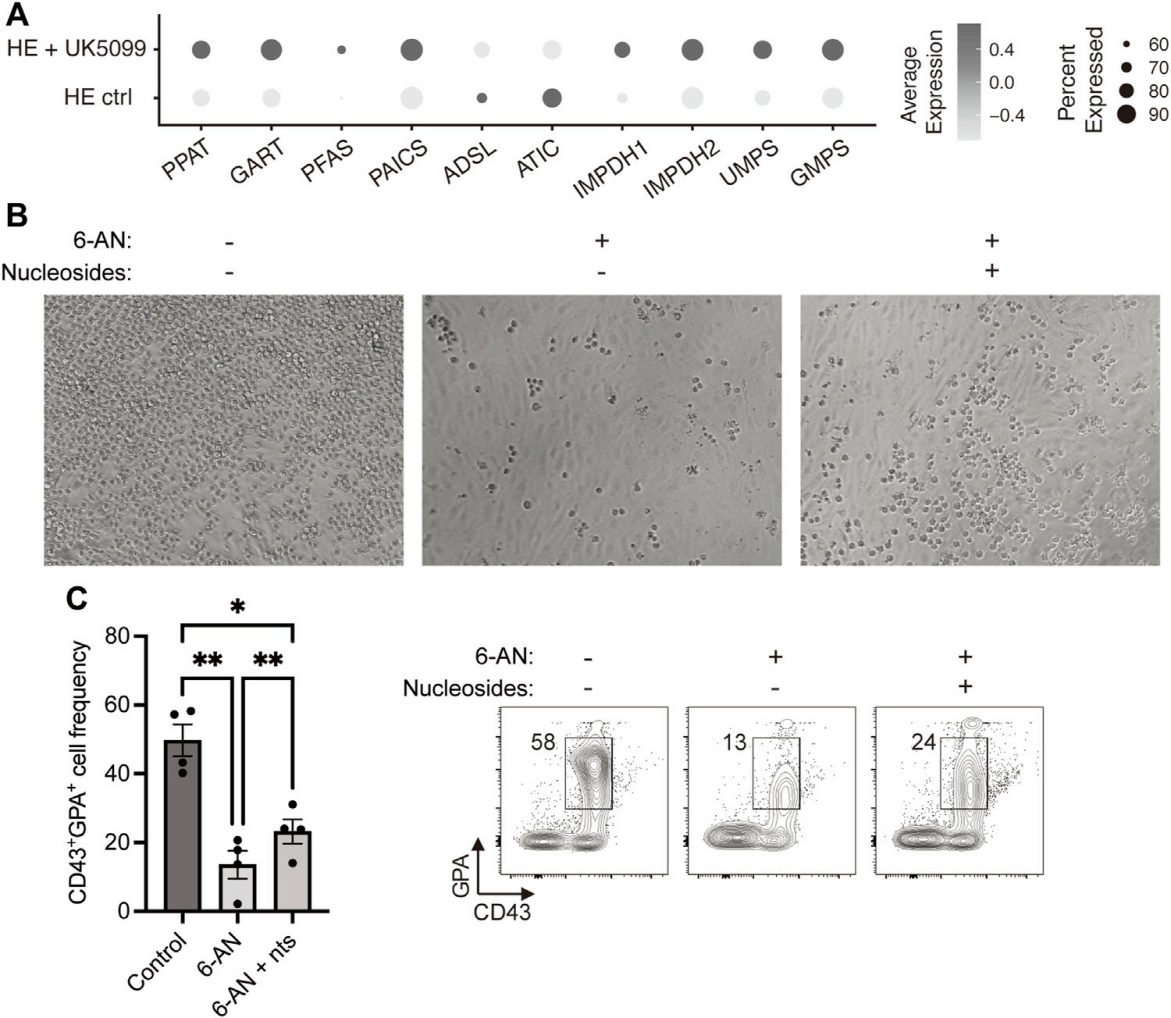
Nucleosides partially rescue 6-AN-mediated block in HE-derived erythroid differentiation. **(A)** Dot plots show expression levels of nucleotide biosynthesis pathway enzymes for HE ctrl and HE + UK5099 in hematopoietic clusters from our previously published dataset, as detected by scRNAseq and based on percent expressed (dot size) and average level of expression (color intensity). **(B,C)** iPSC-derived HE cells were subcultured with or without 25 µM 6-AN and with or without nucleosides (nts) for 6 days. Representative brightfield pictures of the wells **(B)** and representative plots and bar graphs showing CD43^+^GPA^+^ cell frequency ± SEM at day 6 **(C)** are presented (*n* = 3 with 1-2 technical replicates, paired *t*-tests). Culture media was changed every 2 days and inhibitors were added at every media change. ns, not significant, *p < 0.05, **p < 0.01, ****p < 0.0001.

Using our previously published scRNAseq dataset, we then investigated the requirement for the nucleotide biosynthetic pathway in primitive erythroid cells and found that 8 out of 10 enzymes of this pathway were upregulated following UK5099 treatment of HE cells (Figure 2A). This result prompted us to evaluate whether PPP-derived nucleotides are required for developmental erythropoiesis. We treated HE cells with either 6-AN alone or in combination with nucleosides (a cell-permeable form of nucleotides) and found that while 6-AN treatment abrogated the formation of round blood cells from HE, the addition of nucleosides partially restored their production (Figure 2B). Specifically, we observed >75% increase in the frequency of CD43^+^GPA^+^ cells at day 6 in the 6-AN + nucleosides condition, compared to 6-AN treatment alone (Figure 2C), corresponding to >25% rescue with nucleotides. Taken together, these results show that the PPP is an essential metabolic pathway in HE-derived erythroid differentiation and fuels nucleotide biosynthesis and proliferation.

### FOXO1 inhibition boosts hemogenic endothelium-derived erythroid differentiation *via* the pentose phosphate pathway

**FIGURE 3:**
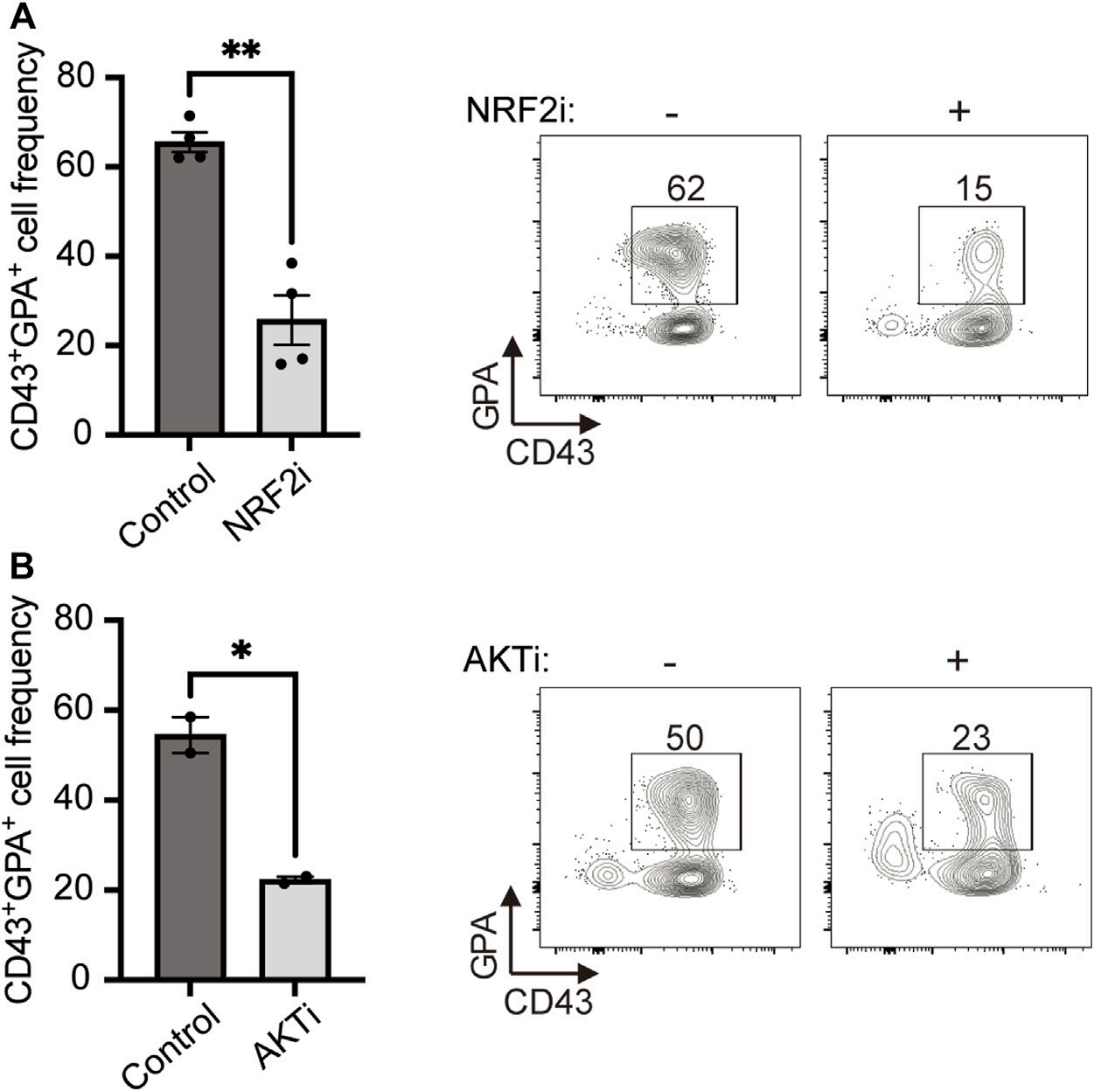
HE-derived erythroid cell differentiation requires NRF2 and AKT. **(A,B)** iPSC-derived HE cells were subcultured with NRF2i **(A)** or AKTi **(B)** for 6 days and compared to untreated cells. Representative plots and bar graphs showing CD43^+^GPA^+^ cell frequency ± SEM at day 6 are presented (*n* = 4 for NRF2i and *n* = 2 for AKTi, paired *t*-tests). Culture media was changed every 2 days and inhibitors were added at every media change. *p < 0.05, **p < 0.01.

Next, we sought to uncover the regulation of PPP in HE-derived erythroid cells. Previously, the transcription factor NRF2 (nuclear factor erythroid 2-related factor 2) has been shown to induce both nucleotide biosynthesis and the PPP (Wu et al., 2011; Mitsuishi et al., 2012; Hawkins et al., 2016; Fox et al., 2020). Therefore, we investigated whether NRF2 plays a role in HE-derived erythropoiesis by blocking this transcription factor with a specific inhibitor (NRF2i). We found that NRF2i treatment led to a 2.6-fold decrease in CD43^+^GPA^+^ cell output (Figure 3A) while the frequency of CD43^+^CD45^+^ cells was not affected (Supplementary Figure S2A), confirming an essential role for this transcription factor in erythroid differentiation from HE. As NRF2 has been shown to be controlled by AKT, the master-regulator of cell proliferation and metabolism (Nakaso et al., 2003; Taguchi et al., 2014; Lien et al., 2016), we also investigated its role in our model. Blocking AKT with a specific inhibitor (AKTi) severely decreased erythroid cell output from HE cells (Figure 3B) while the frequency of CD43^+^CD45^+^ cells was not affected (Supplementary Figure S2B), suggesting that AKT also plays a role in inducing PPP and nucleotide biosynthesis in erythroid specification.

**FIGURE 4:**
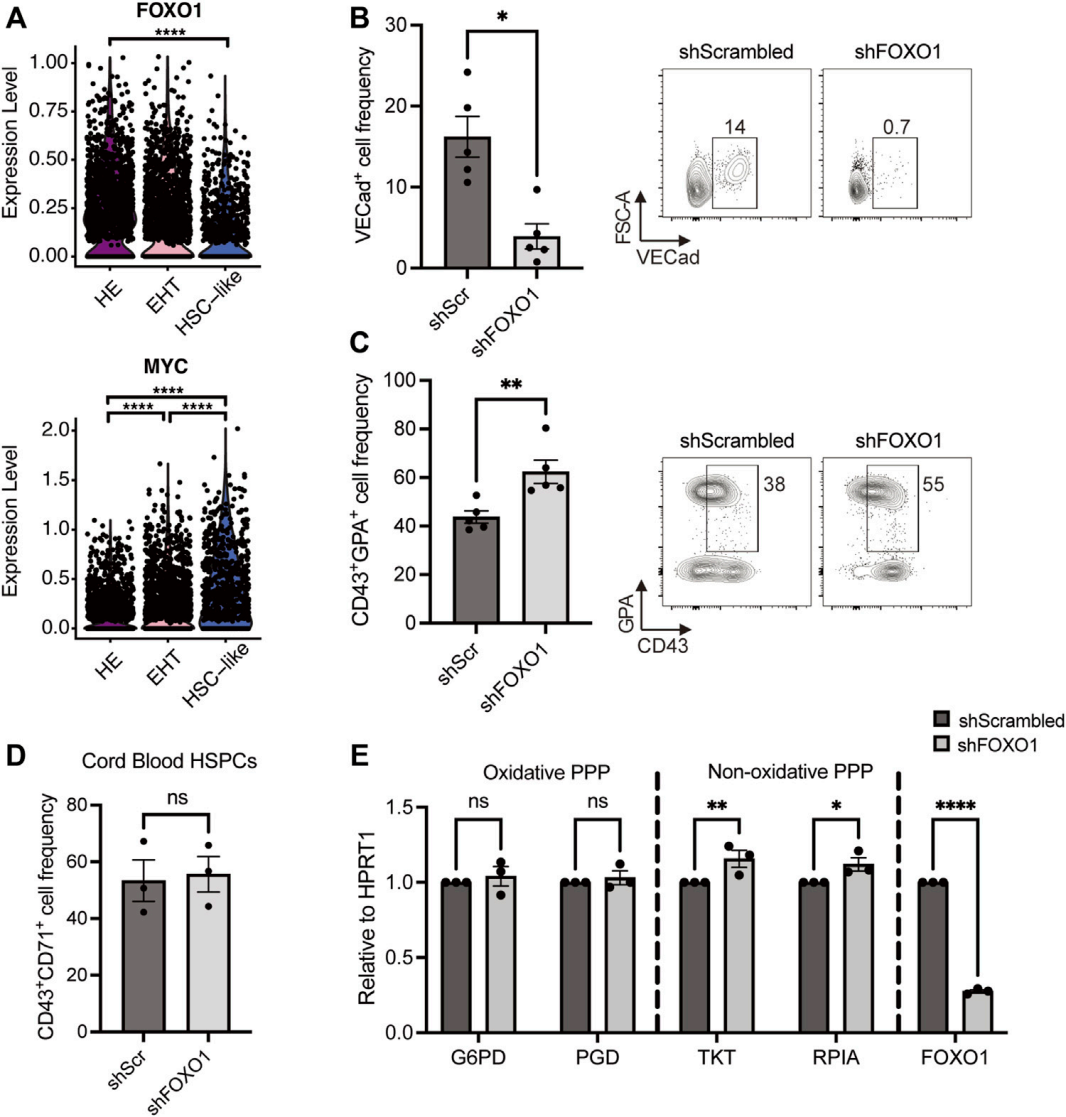
FOXO1 knockdown increases HE-derived erythroid cell output and upregulates the non-oxidative pentose phosphate pathway. **(A)** Violin plots show expression levels of *FOXO1* and *MYC* for HE, EHT and HSC-like cells from our previously published dataset, as detected by scRNAseq (Negative binomial regression tests). **(B,C)** iPSC-derived HE cells were transduced with lentivirus with shRNAs targeting *FOXO1* or a control scrambled shRNA (shScr) and subcultured for 6 days. Representative plots and bar graphs showing VECad^+^ cell frequency ± SEM **(B)** CD43^+^GPA^+^ cell frequency ± SEM **(C)** at day 6 are presented (*n* = 5, paired *t*-tests). **(D)** Umbilical cord blood-derived HSPCs were transduced with the indicated shRNA-harboring lentivirus 2 days after isolation. Bar graphs show CD43^+^CD71^+^ cell frequency ± SEM after 3 days of erythroid differentiation (*n* = 3, paired *t*-tests). **(E)** iPSC-derived HE cells were transduced with lentivirus with shFOXO1 or shScr and subcultured for 3 days. Day 3 transcript levels of pentose phosphate pathway enzymes relative to *HPRT1* are shown (*n* = 3, 2-way ANOVA). ns, not significant, *p < 0.05, **p < 0.01, ****p < 0.0001.

In previous studies, AKT has been shown to regulate cell proliferation and differentiation by repressing FOXO transcription factors (Brunet et al., 1999; Bouchard et al., 2004; Zhang et al., 2011), namely in hematopoietic cells (Ochiai et al., 2012; Shyh-Chang et al., 2013; Clark et al., 2014). Moreover, the FOXO1 transcription factor has been shown to preserve the quiescent state of endothelial cells by repressing the expression of MYC (Wilhelm et al., 2016). This prompted us to investigate whether FOXO1 and MYC also play similar roles in hemogenic endothelial cells. Gene expression profiles from our previously published scRNAseq dataset show that HE cells express the highest levels of *FOXO1* when compared with EHT and HSC-like cells, and *FOXO1* expression gradually decreases during EHT (Figure 4A). In contrast, *MYC* levels are lowest in HE cells and increase during EHT. This data suggested a role for the FOXO1-MYC axis in HE cells. To investigate this, we downregulated either *FOXO1* or *MYC* in HE cells using shRNAs (efficiencies shown in Supplementary Figures S1A,B, respectively) and assessed erythroid differentiation 6 days later. Intriguingly, *FOXO1* downregulation led to a 4-fold decrease in the frequency of VECad^+^ endothelial cells (Figure 4B), while *MYC* downregulation did not result in any changes (Supplementary Figure S1C). In contrast, downregulating *FOXO1* significantly increased CD43^+^GPA^+^ cell frequency (Figure 4C), while shRNA against MYC once again did not have an effect (Supplementary Figure S1D). It is important to note that *FOXO1* downregulation did not have an effect on *MYC* transcript levels in this context (Supplementary Figure S1E), suggesting that the effect of FOXO1 on erythroid differentiation could be independent of MYC.

In order to understand whether the FOXO1 effect we observed in HE-derived erythroid differentiation is specific to developmental hematopoiesis, we investigated the role of *FOXO1* during neonatal erythroid differentiation, namely in UCB-derived CD34^+^ HSPCs. As the GPA marker appears late during the differentiation and does not inform on the maturity of the erythroid cell, we focused on the expression of CD71 (transferrin receptor) which is expressed at an earlier stage on erythroid cells (Hu et al., 2013). Interestingly, at an early time point (day 3) when only around 50% of control cells express CD71, *FOXO1* downregulation did not lead to an increase in CD43^+^CD71^+^ erythroid progenitors deriving from UCB HSPCs (Figure 4D), suggesting that the regulation by *FOXO1* is specific to HE-derived erythropoiesis. We then assessed whether *FOXO1* directly controls the induction of PPP and measured the expression of several enzymes of this pathway after shFOXO1 treatment in HE cells. We found that shFOXO1 treatment did not change the expression levels of oxidative PPP enzymes *G6PD* and *PGD* (Figure 4E, Oxidative PPP). However, *FOXO1* downregulation led to a slight but significant increase in the expression of *TKT* and *RPIA*, two main enzymes of the non-oxidative branch of the PPP (Figure 4E, Non-oxidative PPP). All together, these results indicate that by modulating the non-oxidative branch of the PPP, FOXO1 restricts erythroid differentiation of HE cells during developmental hematopoiesis.

## Discussion

In this study, we showed that human HE-derived erythroid differentiation is dependent on the PPP. We found that the PPP fuels proliferation and nucleotide biosynthesis during the formation and expansion of erythroid cells (Supplementary Figure S3). Previously, we have shown that both the PPP and nucleotide biosynthesis are instrumental for erythroid differentiation of UCB HSPCs (Oburoglu et al., 2014). Other metabolic changes are required as erythroid cells mature: while terminal maturation relies on decreasing α-ketoglutarate-dependent oxidative phosphorylation (Gonzalez-Menendez et al., 2021), enucleation is fueled by pyruvate which maintains a low mitochondrial activity level in erythroblasts (Liang et al., 2021).

Interestingly, in the present study, we observed that the PPP and erythroid differentiation are regulated by FOXO1 specifically during developmental erythropoiesis, but not in UCB HSPC-derived progenitors. In a seminal study, FOXO1 was shown to regulate metabolic processes in endothelial cells (Wilhelm et al., 2016) and similarly, here we observed that FOXO1 was highly expressed in HE cells but its expression gradually decreased during EHT and hematopoietic differentiation. This suggests that FOXO1 regulates the PPP specifically in endothelial cells and this does not persist as the cells differentiate to the hematopoietic lineage. Moreover, even though we could not observe an effect of *FOXO1* knockdown on *MYC* mRNA levels, it will be of interest in future studies to determine whether there is an interplay between FOXO1 and MYC in this context.

We observed here that NRF2 is essential for HE-derived erythroid differentiation. The transcription factor NRF2 is a well-described player in combating reactive oxygen species (ROS) and oxidative stress (Nakaso et al., 2003; DeNicola et al., 2011; Fox et al., 2020). Moreover, NRF2 can act by boosting PPP-derived NADPH, which is a ROS-scavenger (Wu et al., 2011; Hawkins et al., 2016). The production of NADPH occurs *via* the oxidative branch of the PPP, specifically during the oxidation of glucose 6-phosphate into 6-phosphogluconolactone by G6PD (Riganti et al., 2012). We show here that FOXO1 inhibition does not induce enzymes of the oxidative branch of PPP. Thus, it will be of interest to understand in future studies whether NRF2 induces the activation of the NADPH-producing oxidative branch of PPP in HE-derived erythropoiesis. Since our data showed that nucleotides were not sufficient to completely rescue the effect of PPP inhibition during erythroid differentiation of HE cells, studying the role of NADPH production could provide additional insights into this matter.

Both NRF2 and FOXO1 are controlled by the master regulator of cell survival AKT (Nakaso et al., 2003; DeNicola et al., 2011; Koundouros and Poulogiannis, 2018) and indeed, we showed here that AKT is crucial for the induction of erythropoiesis from HE cells. In several cell types, including hematopoietic cells, AKT has been shown to repress FOXO (Brunet et al., 1999; Bouchard et al., 2004; Ochiai et al., 2012; Clark et al., 2014). Thus, our results strongly suggest that complementarily to the known NRF2 activation, by repressing FOXO1, AKT induces erythroid differentiation of HE cells.

Importantly, we showed here that FOXO1 repression induces a small-scale increase in the expression of non-oxidative PPP enzymes *RPIA* (ribose 5-phosphate isomerase A) and *TKT* (transketolase). Accordingly, constitutively active FOXO1 expression in the liver has been shown to specifically downregulate the expression of ribose 5-phosphate isomerase and transketolase in transgenic mice (Zhang et al., 2006). This specific non-oxidative branch of PPP produces fructose 6-phosphate and glyceraldehyde 3-phosphate, which can either be reused to re-fuel PPP or enter the glycolytic pathway (Riganti et al., 2012). Indeed, we and others have previously shown that glycolysis is essential for erythroid differentiation (Oburoglu et al., 2014; Goto et al., 2019; Oburoglu et al., 2022). Thus, an increase in glycolysis *via* non-oxidative PPP could explain the boost in erythroid differentiation we observed in FOXO1-deficient cells.

Taken together, our results provide insight into the role of PPP in developmental erythropoiesis (Supplementary Figure S3). High FOXO1 levels in HE cells prevent their differentiation into erythroid cells; however, with the gradual decrease in FOXO1 expression during EHT and hematopoietic differentiation, the PPP is activated and contributes to nucleotide biosynthesis and possibly glycolysis, which in turn fuel the rapid proliferation and differentiation of erythroid progenitors.

## Materials and methods

### Human iPSC culture and erythroid differentiation

The human iPSC line RB9-CB1 (Woods et al., 2011) was used in all experiments and hematopoietic differentiation was induced as described previously (Ditadi and Sturgeon, 2016; Oburoglu et al., 2022). Briefly, iPSCs were maintained as colonies on mouse embryonic fibroblasts (MEFs) for 6 days, after which embryoid bodies (EBs) were set up. Hematopoietic differentiation was induced for 8 days, at which point the EBs were dissociated with TryPLE Express (Thermo Fisher Scientific) and processed to positively select CD34^+^ cells using magnetic beads (human CD34 MicroBead kit, Miltenyi Biotec). In experiments where only HE was assessed, the selected CD34^+^ cells were plated onto Matrigel (16 μg/cm^2^, Corning)-coated 96-well flat bottom plates and supplemented with HE medium (Ditadi and Sturgeon, 2016) with 1% penicillin-streptomycin and kept in a humidified incubator at 37°C, 5% CO_2_. The next day (day 0), blood cells were washed away twice with PBS and endothelial cells were kept in HE medium with 1% penicillin-streptomycin in a humidified incubator at 37°C, 5% CO_2_ for 6 more days to induce erythroid differentiation. At day 0, 25 µM 6-aminonicotimamide (6-AN, Merck-Sigma Aldrich)**,** Nucleosides (Cytidine: 7.3 mg/L; Guanosine: 8.5 mg/L; Uridine: 7.3 mg/L; Adenosine: 8 mg/L; Thymidine: 2.4 mg/L; EmbryoMax, Merck Millipore), 5 µM AKTi (Akti1/2, Merck-Sigma Aldrich) or 5 µM NRF2i (ML385, Merck-Sigma Aldrich) was added to the medium. Media was supplemented with the aforementioned reagents every 2 days until day 6. In experiments where HE, EHT and HSC-like cells were assessed, the CD34^+^ cells were selected after 10 days of hematopoietic differentiation, the HE (CD34^+^CD43^−^CXCR4^−^CD73^−^CD90^+^VECad^+^), EHT (CD34^+^CD43^int^CXCR4^−^CD73^−^CD90^+^VECad^+^) and HSC-like (CD34^+^CD43^+^CD90^+^CD38^−^) cells were sorted based on markers described previously (Choi et al., 2012; Ditadi et al., 2015; Guibentif et al., 2017) and kept in culture as indicated above.

### Flow cytometry analyses and cell sorting

On day 6 of subculture, HE-derived cells were collected with StemPro Accutase Cell Dissociation Reagent and stained with GPA-PE, CD45-AF700, CD43-APCH7 and the viability marker DAPI and analysed on a BD LSRII. Proliferation was assessed with the CellTrace Violet (CTV) kit according to manufacturer’s instructions. Briefly, 5 µM CTV was used to stain the cells during a 10-min incubation at 37°C. Fluorescence was measured on a BD LSRFortessa. In experiments where HE, EHT and HSC-like cells were assessed, the selected iPSC-derived CD34^+^ cells were stained with CD34-FITC, CD73-PE, VECad-PerCPCy5.5, CD38-PC7, CD184-APC, CD45-AF700, CD43-APCH7, GPA-eF450, CD90-BV605 and the viability marker 7AAD. The HE (CD34^+^CD43^−^CXCR4^−^CD73^−^CD90^+^VECad^+^), EHT (CD34^+^CD43^int^CXCR4^−^CD73^−^CD90^+^VECad^+^) and HSC-like (CD34^+^CD43^+^CD90^+^CD38^−^) cells were sorted on a BD FACSARIA according to the indicated markers and following the sorting strategies described previously (Oburoglu et al., 2022). In experiments where GPA^+^ cells or CD45^+^ cells were assessed, viable cells expressing either marker were sorted at day 13 of the hematopoietic differentiation protocol on a BD FACSARIA. For all experiments, flow cytometry results were analysed on the FlowJo Software, with gatings on SSC-A/FSC-A, FSC-H/FSC-A, SSC-H/SSC-A to exclude doublets and DAPI to exclude dead cells.

### shRNA-mediated gene knockdown

Short-hairpin RNA sequences designed to recognize the genes of interest (FOXO1, TRCN0000039580 and MYC, TRCN0000039642; from MERCK) were cloned into GFP-expressing pRRL-SFFV vectors, embedded in a microRNA context, as described previously (Fellmann et al., 2013). Lentiviral particles were produced in HEK 293T cells plated in T175 flasks by co-transfection of 22 μg of pMD2.G, 15 μg of pRSV-Rev, 30 μg of pMDLg/pRRE and 75 μg of the shRNA vector using 2.5 M CaCl_2_. Sixteen hours after transfection, media was changed and viruses were harvested 24 h later. Viral particles were pelleted at 20,000 x g for 2 h at 4°C, resuspended in 100 μl DMEM and kept at -80°C. The efficiency of each shRNA was measured by lentiviral transduction of UCB CD34^+^ HSPCs and assessment of the corresponding gene expression by qPCR in sorted Day 3 GFP^+^ cells (see Supplementary Figures S1A,B).

### Isolation of cord blood hematopoietic stem and progenitor cells, transduction and erythroid differentiation

Umbilical cord blood samples were collected after informed consent and approval by the regional ethical committee at Lund University, at Skåne University Hospital in Lund and Malmö or at Helsingborg General Hospital, Sweden. The samples were processed after 1:1 dilution with PBS (1X) by density gradient centrifugation using Lymphoprep tubes (Serumwerk, Bernburg, Germany) to isolate mononuclear cells. Next, CD34^+^ enrichment was performed using the human CD34 MicroBead kit (Miltenyi Biotec), with two consecutive columns to increase HSPC purity. Isolated CD34^+^ cells were expanded in Stemspan SFEM medium (Stem cell technologies) supplemented with 5% fetal bovine serum (Thermo Fisher Scientific), 25 ng/ml stem cell factor (SCF), 10 ng/ml IL-6 and 10 ng/ml IL-3 (Peprotech) for 2 days before lentiviral transduction. The day after transduction, early erythroid differentiation was induced by addition of 3 IU/ml EPO (Retacrit, Hospira UK Ltd.) to the medium, following previously described protocols (Oburoglu et al., 2014; Gonzalez-Menendez et al., 2021).

### Statistical analyses

The statistical analyses used to determine the significance of differences between conditions were paired *t*-tests or 2-way analysis of variance (ANOVA) tests with multiple comparisons, as indicated. The GraphPad Prism 6 software was used to perform the analyses and *p* values are indicated in figures with the following abbreviations: ns, not significant, *p < 0.05, **p < 0.01, ***p < 0.001, ****p < 0.0001.

## Data Availability

The original contributions presented in the study are included in the article and Supplementary Material (Supplementary Table S1); further inquiries can be directed to the corresponding author.
