# Supplementary material for: FOXO1 regulates pentose phosphate pathway-mediated induction of developmental erythropoiesis
Source: Front Cell Dev Biol. 2022 Oct 12;10:1039636. doi: 10.3389/fcell.2022.1039636 (PMC9596918; doi:10.3389/fcell.2022.1039636)
Supplement: Supplementary file 1 [file Table1.docx]

**Reagent table**

| **REAGENT** | **SOURCE** | **IDENTIFIER** |
| --- | --- | --- |
| **Taqman probes** |  |  |
| G6PD | Thermo Fisher Scientific | Hs00166169_m1 |
| PGD | Thermo Fisher Scientific | Hs00427230_m1 |
| TKT | Thermo Fisher Scientific | Hs01115545_m1 |
| RPIA | Thermo Fisher Scientific | Hs01107136_m1 |
| FOXO1 | Thermo Fisher Scientific | Hs00231106_m1 |
| MYC | Thermo Fisher Scientific | Hs00153408_m1 |
| HPRT1 | Thermo Fisher Scientific | Hs02800695_m1 |
| **Biological Samples** |  |  |
| Human Umbilical Cord Blood samples (informed consents according to guidelines approved by the regional ethical committee) | Skåne University Hospital (Lund and Malmö) and Helsingborg Hospital | N/A |
| **Antibodies (anti-human)** | | |
| CD43- Clone 1G10 | BD Biosciences | Cat# 655407 |
| CD34- Clone 561 | Biolegend | Cat# 343604; RRID:AB_1732005 |
| CD144 (VE-Cadherin)- Clone 55-7H1BD | BD Biosciences | Cat# 561566; RRID:AB_10715835 |
| CD73- Clone AD2 | BD Biosciences | Cat# 550257; RRID:AB_393561 |
| GPA- Clone HIR2 (GA-R2) | Thermo Fisher Scientific | Cat# 12-9987-82; RRID: AB_466300 |
| CD45- Clone HI30 | Biolegend | Cat# 304024; RRID:AB_493761 |
| CD90- Clone 5E10 | BD Biosciences | Cat# 562685; RRID:AB_2744468 |
| CD38- Clone HIT2 | Biolegend | Cat# 303516; RRID:AB_2072782 |
| CD71- Clone M-A712 | BD Biosciences | Cat# 561940; RRID:AB_10898005 |
| CD45- Clone HI30 | Biolegend | Cat# 304024; RRID:AB_493761 |
| CD184 (CXCR4)- Clone 12G5 | BD Biosciences | Cat# 555976; RRID:AB_398616 |
| **Chemicals, Peptides, and Recombinant Proteins** | | |
| 6-Aminonicotinamide (6-AN) | Sigma-Aldrich | Cat# A68203 |
| Akti-1/2 (AKT Inhibitor) | Sigma-Aldrich | Cat# 124017 |
| ML385 (NRF2 Inhibitor) | Sigma-Aldrich | Cat# SML1833 |
| EmbryoMax Nucleosides (100X) | Sigma-Aldrich | Cat# ES-008-D |
| MEM Non-Essential Amino Acids Solution (100X) | Thermo Fisher Scientific | Cat# 11140050 |
| DAPI (4',6-Diamidino-2-Phenylindole, Dilactate) | Biolegend | Cat# 422801 |
| CHIR99021 | R&D Systems | Cat# 4423/10 |
| Activin A | R&D Systems | Cat# 338-AC |
| StemPro Accutase Cell Dissociation Reagent | Thermo Fisher Scientific | Cat# A1110501 |
| Hyclone Penicillin-Streptomycin Solution | Thermo Fisher Scientific | Cat# SV30010 |
| GlutaMAX Supplement | Thermo Fisher Scientific | Cat# 35050038 |
| Recombinant Human SCF | Peprotech | Cat# 300-07 |
| Recombinant Human IL6 | Peprotech | Cat# 200-06 |
| Recombinant Human BMP4 | Peprotech | Cat# AF-120-05ET |
| Recombinant Human VEGF 165 | Peprotech | Cat# AF-100-20 |
| Recombinant Human GM-CSF | Peprotech | Cat# 300-03 |
| Recombinant Human IL-3 | Peprotech | Cat# 200-03 |
| Recombinant Human FGF-basic | Peprotech | Cat# 100-18B |
| Recombinant Human Flt3-L | Peprotech | Cat# 300-19 |
| Recombinant Human IGF-I | Peprotech | Cat# 100-11 |
| Recombinant Human IL-11 | Peprotech | Cat# AF-200-11 |
| Retacrit 10 000 IU/1 mL solution (EPO) | Hospira UK Ltd |  |
| Stemspan SFEM | Stemcell Technologies | Cat# 09650 |
| DMEM-F12 | Thermo Fisher Scientific | Cat# 31330 |
| StemPro-34 SFM (1X) | Thermo Fisher Scientific | Cat# 10639011 |
| Ascorbic Acid | Sigma-Aldrich (Merck) | Cat# A4544 |
| 2-Mercaptoethanol (50 mM) | Thermo Fisher Scientific | Cat# 31350010 |
| N2 (100x) | Thermo Fisher Scientific | Cat# 17502048 |
| B27 minus VitA (50x) | Thermo Fisher Scientific | Cat# 12587010 |
| holo-Transferrin human | Sigma-Aldrich (Merck) | Cat# T0665 |
| Losartan potassium | Tocris (R&D Systems) | Cat# 3798 |
| Angiotensin II human | Sigma-Aldrich (Merck) | Cat# A9525 |
| Recombinant Human Sonic Hedgehog/Shh | R&D Systems | Cat# 1314-SH-025/CF |
| FBS | Thermo Fisher Scientific | Cat# SV30160 |
| Matrigel Matrix | Corning | Cat# 354230 |
| TrypLE Express | Thermo Fisher Scientific | Cat# 12604-013 |
| HyClone Phosphate Buffered Saline (PBS) | Thermo Fisher Scientific | Cat# 10462372 |
| **Critical Commercial Assays** | | |
| CD34 MicroBead Kit, human | Miltenyi Biotec | Cat# 130-046-703 |
| CellTrace Violet Cell Proliferation Kit | Thermo Fisher Scientific | Cat# C34571 |
| \| RNeasy Mini Kit \|  \|  \| \| --- \| --- \| --- \| | QIAGEN | Cat# 74104 |
| TaqMan Gene Expression Master Mix | Thermo Fisher Scientific | Cat# 4369542 |
| NucleoBond Xtra Midi prep kit | Macherey-Nagel | Cat# 740410.50 |
| **Experimental Models: Cell Lines** | | |
| iPSC-CB1RB9 cell line, human (RB9-CBiPS2) | Woods et al, STEM CELLS (2011) | PMID: 21544903 |
| Mouse Embryonic Fibroblasts | Merck-Millipore | Cat# PMEF-NL |
| **Software and Algorithms** | | |
| FlowJo | BD Life Sciences | https://www.flowjo.com/solutions/flowjo/ downloads |
| GraphPad Prism 6 | GraphPad | http://www.graphpad.com/support/faqid/%201952 |
| FACSDiva v8.0.1 | BD Bioscience | https://www.bdbiosciences.com/en-us/instruments/research-instruments/research-software/flow-cytometry-acquisition/facsdiva-software |
| Adobe Illustrator CS6 | Adobe Systems Inc | https://www.adobe.com/cn/products/cs6/illustrator.html |
| Fiji | Image J | https://imagej.net/Fiji/Downloads |
| RStudio (v1.2.1578) |  | https://rstudio.com/about/ |
| Seurat package v3.1.0 | Butler et al, Nat Biotech (2018); Stuart et al, Cell (2019) | PMID: 29608179  PMID: 31178118 |
| Adobe Illustrator CS6 | Adobe Systems Inc | https://www.adobe.com/cn/products/cs6/illustrator.html |
