# Supplementary material for: FOXO1 regulates pentose phosphate pathway-mediated induction of developmental erythropoiesis
Source: Front Cell Dev Biol. 2022 Oct 12;10:1039636. doi: 10.3389/fcell.2022.1039636 (PMC9596918; doi:10.3389/fcell.2022.1039636)
Supplement: Supplementary file 2 [file DataSheet1.docx]

**
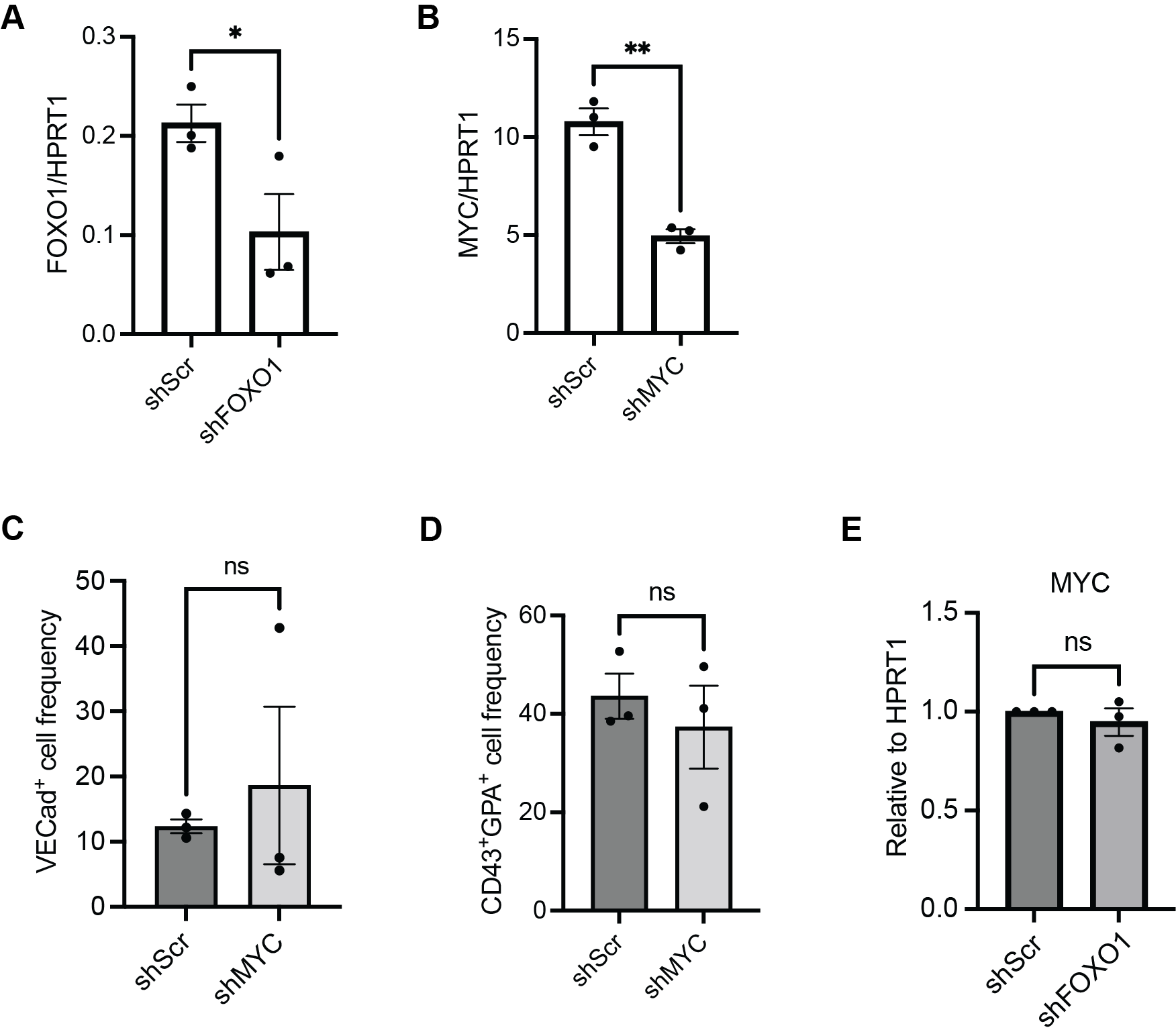
**

**Figure S1. Effects of *FOXO1* and *MYC* downregulation in shRNA-treated cells**

**(A-B)** Umbilical cord blood-derived HSPCs were transduced with the indicated shRNA-harboring lentiviruses 2 days after isolation. After a 3-day expansion, GFP^+^ cells were sorted by flow cytometry and *FOXO1* **(A)** and *MYC* **(B)** transcript levels relative to *HPRT1* are shown (n=3, paired *t*-tests). **(C-D)** iPSC-derived HE cells were transduced with lentivirus with shRNAs targeting *MYC* or a control scrambled shRNA (shScr) and subcultured for 6 days. Representative plots and bar graphs showing VECad^+^ cell frequency ± SEM **(C)** CD43^+^GPA^+^ cell frequency ± SEM **(D)** at day 6 are presented (n=3, paired *t*-tests). **(E)** iPSC-derived HE cells were transduced with the indicated shRNA-harboring lentiviruses and subcultured for 3 days. Day 3 *MYC* transcript levels relative to *HPRT1* are shown (n=3, paired *t*-tests). ns, not significant, *p<0.05, **p<0.01


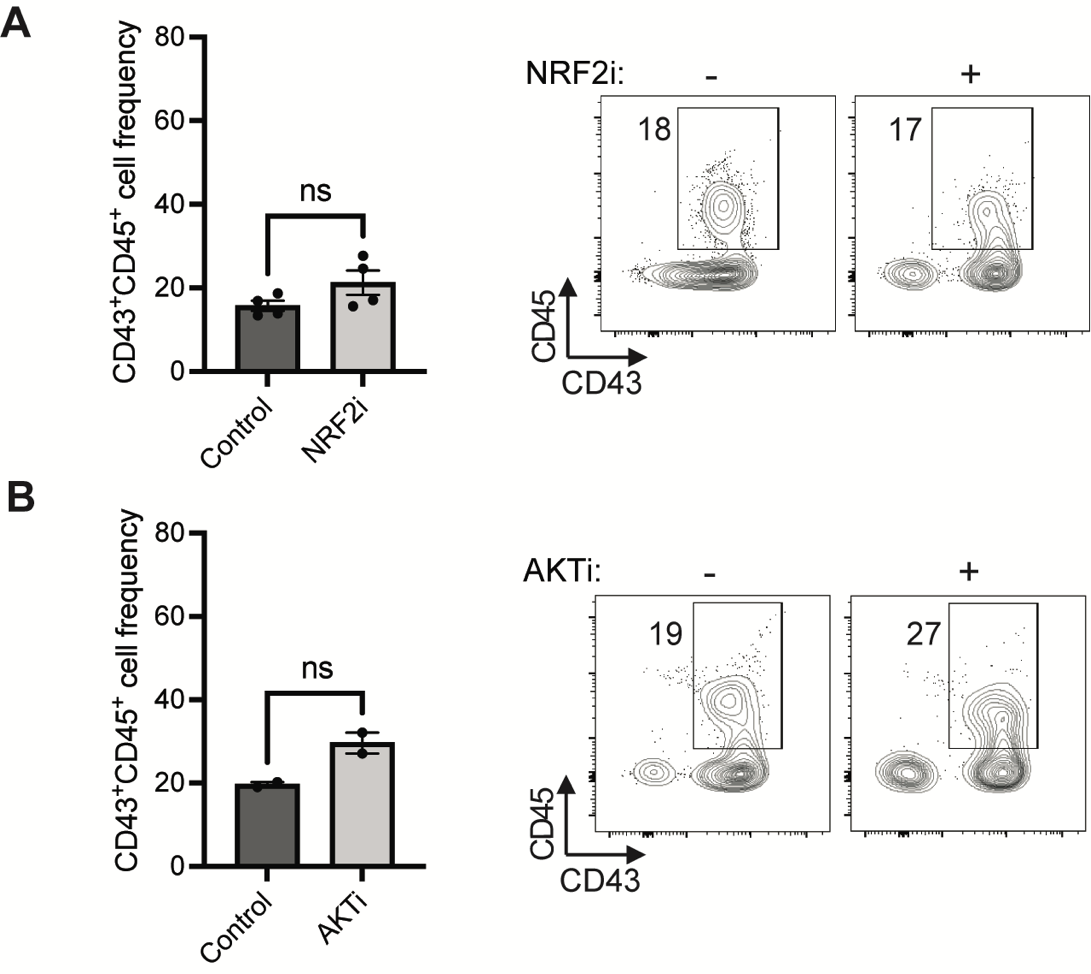


**Figure S2. HE-derived CD43^+^CD45^+^ frequencies are not affected by blocking NRF2 or AKT**

**(A-B)** iPSC-derived HE cells were subcultured with NRF2i **(A)** or AKTi **(B)** for 6 days and compared to untreated cells. Representative plots and bar graphs showing CD43^+^CD45^+^ cell frequency ± SEM at day 6 are presented (n=4 for NRF2i and n=2 for AKTi, paired *t*-tests). Culture media was changed every 2 days and inhibitors were added at every media change. ns, not significant


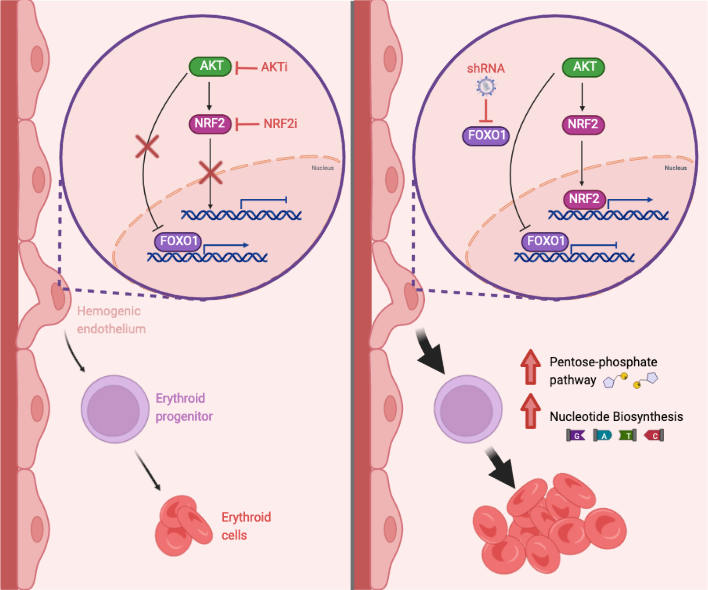


**Figure S3. FOXO1 regulates pentose phosphate pathway-mediated induction of developmental erythropoiesis**

We show here that erythroid differentiation of hemogenic endothelium is dependent on AKT and NRF2. We uncover that the pentose phosphate pathway is regulated by FOXO1 and controls nucleotide biosynthesis and erythroid cell proliferation. We find that this regulatory mechanism is specific to developmental erythropoiesis. Created with BioRender.com.
